# Glycomacropeptide Ameliorates Indomethacin-Induced Enteropathy in Rats by Modifying Intestinal Inflammation and Oxidative Stress

**DOI:** 10.3390/molecules25102351

**Published:** 2020-05-18

**Authors:** Daniel Cervantes-García, Armida I. Bahena-Delgado, Mariela Jiménez, Laura E. Córdova-Dávalos, Vanessa Ruiz-Esparza Palacios, Esperanza Sánchez-Alemán, María C. Martínez-Saldaña, Eva Salinas

**Affiliations:** 1 Department of Microbiology, Basic Science Center, Autonomous University of Aguascalientes, Aguascalientes 20131, Mexico; cervantes.daniel@gmail.com (D.C.-G.); armi.bade@gmail.com (A.I.B.-D.); mayojv@hotmail.com (M.J.); lcdavalos@gmail.com (L.E.C.-D.); vane_ruiz.28@hotmail.com (V.R.-E.P.); 2 National Council of Science and Technology, Mexico City 03940, Mexico; 3 Department of Morphology, Basic Science Center, Autonomous University of Aguascalientes, Aguascalientes 20131, Mexico; espesanchez@correo.uaa.mx (E.S.-A.); mcmtzsal@correo.uaa.mx (M.C.M.-S.); 4 Unit of Familiar Medicine #8, Mexican Institute of Social Security, Aguascalientes 20180, Mexico

**Keywords:** nonsteroidal anti-inflammatory drugs, intestinal damage, glycomacropeptide, neutrophil infiltration, inflammatory mediators, oxidative damage, mucosal barrier integrity

## Abstract

Nonsteroidal anti-inflammatory drug (NSAID)-induced enteropathy is considered a serious and increasing clinical problem without available treatment. Glycomacropeptide (GMP) is a 64-amino acid peptide derived from milk κ-casein with numerous biological activities. The aim of this study was to investigate the protective effect of GMP on NSAID enteropathy in rats. Enteropathy was induced by seven days oral indomethacin administration. Rats were orally GMP treated from seven days previous and during the establishment of the enteropathy model. Changes in metabolism, hematological and biochemical blood alterations, intestinal inflammation and oxidative damage were analyzed. Integrity barrier markers, macroscopic intestinal damage and survival rate were also evaluated. GMP treatment prevented anorexia and weight loss in animals. Furthermore, prophylaxis with GMP ameliorated the decline in hemoglobin, hematocrit, albumin and total protein levels. The treatment had no therapeutic efficacy on the decrease of occludin and mucin (MUC)-2 expression in intestinal tissue. However, GMP markedly decreased neutrophil infiltration, and CXCL1, interleukin-1β and inducible nitric oxide synthase expression. Nitric oxide production and lipid hydroperoxide level in the small intestine were also diminished. These beneficial effects were mirrored by preventing ulcer development and increasing animal survival. These results suggest that GMP may protect against NSAID enteropathy through anti-inflammatory and antioxidant properties.

## 1. Introduction

Nonsteroidal anti-inflammatory drugs (NSAIDs) are a group of medicines widely indicated to treat inflammation and pain in rheumatic and musculoskeletal disorders, like rheumatoid arthritis and osteoarthritis [1]. Because NSAIDs are easily accessed by most of the population, they are indiscriminately used without medical supervision. It is estimated that worldwide, approximately 30 million people consume NSAIDs every day [2]. These anti-inflammatory drugs inhibit prostaglandin (PG) synthesis by preventing arachidonic acid (AA) from binding to the active site of cyclooxygenase (COX) enzymes [3]. Two isoforms of COX are involved in the initial steps of PG synthesis under different conditions. The expression of the COX-2 isoform is induced once inflammation is established, leading to increased production of proinflammatory PGs [4]. Thus, COX-2 inhibition is the basis of NSAID pharmacological use. Nevertheless, the COX-1 isoform is constitutively expressed in the gastrointestinal tract, kidney and platelets, participating in their homeostasis and proper function. COX-1 catalytic activity is crucial in the maintenance and protection of the gastrointestinal mucosa, which is associated with PGE_2_ synthesis [5,6]. The main adverse effects of NSAIDs are due to COX-1 inhibition and are associated with gastroduodenal and small intestinal damage, like ulcer formation, bleeding and perforation [7,8], accompanied by clinical outcomes such as anemia or peritonitis [9,10,11]. Small intestine damage is on the rise, and capsule endoscopy studies have shown that its prevalence is higher than previously suggested, producing NSAID enteropathy that may occur with clinical or subclinical damage [10,11,12]. In this scope, the development of new therapies to ameliorate or prevent NSAID-induced enteropathy is imperative, particularly for patients with chronic use of NSAIDs.

Experimental evidence about the physiopathogenesis of NSAID-induced enteropathy suggests the participation of various mechanisms. First, the damage is started by deprivation of PGE_2_ through the inhibition of both COX-1 and COX-2, the uncoupling of oxidative phosphorylation after acidic NSAID entry to enterocytes, and the consequent increase in mucosal permeability [13,14,15,16]. Once damage has been initiated, luminal factors such as bile acids, pancreatic juice components, dietary macromolecules and mainly Gram-negative bacteria activate mucosal inflammation [17,18,19]. Then, proinflammatory mediators, neutrophil infiltration, and oxidative stress derived from the triggered inflammatory process exacerbate intestinal damage and worsen lesions [14,19,20]. Rodent models are useful to study the potential beneficial effect of drugs or natural substances in enteropathy caused by NSAIDs, as factors contributing to intestinal damage appear to be similar to those reported in humans [18].

Many bioactive peptides exert important health-promoting activities [21]. Several studies have demonstrated the potentially beneficial effects of milk-derived bioactive peptides, suggesting their use as prophylactic agents to alleviate symptoms of various human diseases [22]. Glycomacropeptide (GMP) is a bioactive peptide of 64 amino acids which is derived from the cleavage of milk κ-casein by chymosin during cheese elaboration or by pepsin during the digestion process [23,24]. Apart from the peptide scaffold, GMP contains a high amount of sugar molecules, the most abundant of which is N-acetyl neuroaminic (sialic) acid [25]. Evidence shows that GMP is safe for human use and not immunogenic [26,27]. Due to the low level of phenylalanine in its amino acid composition, it is used to elaborate nutritional formulations for patients with phenylketonuria [28]. Among the biological properties attributed to GMP, anti-inflammatory and antioxidant activities are relevant to this study. Orally administered GMP is found to be anti-inflammatory in rat models of asthma, atopic dermatitis, colitis and ileitis, preventing extensive damage and downregulating proinflammatory mediators in affected tissues [29,30,31,32,33]. GMP also induces a significant decrease in the production of interleukin (IL)-6, IL-1β, and tumor necrosis factor (TNF)-α by murine bone-marrow-derived dendritic cells in response to lipopolyssacharide (LPS) [34]. Another in vitro study demonstrated that GMP and its papain hydrolysate prevented the generation of reactive oxygen species and induced the production of antioxidant enzymes in H_2_O_2_-damaged RAW264.7 macrophages [35]. Therefore, the bioactive properties of GMP suggest its potential use in the prevention of intestinal damage by NSAID administration. The present study was designed to explore the protective effect of orally administered GMP on NSAID-induced enteropathy in a rat model, focusing on the possible anti-inflammatory and antioxidant activities of GMP.

## 2. Results

### 2.1. Effect of GMP on Food Intake and Body Weight Gain

**Figure 1:**
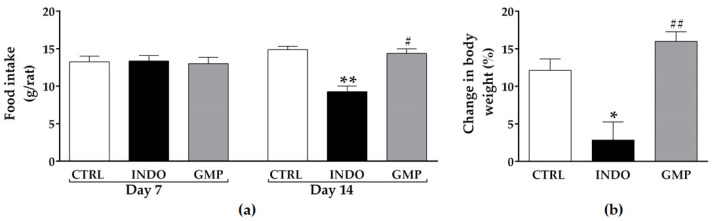
Effect of treatment with glycomacropeptide (GMP) on food intake and body weight gain in rats with indomethacin (INDO)-induced enteropathy. (**a**) Daily food intake of rats at day 7 and 14 of the experimental enteropathy protocol. (**b**) Percentage (%) change in body weight of animals at day 14 in relation to that of day 8. Data are presented as mean ± standard error of the mean (SEM). CTRL and GMP, *n* = 14; INDO, *n* = 11, except day 7 where *n* = 14. * *p* < 0.01 and ** *p* < 0.001 INDO vs. CTRL; # *p* < 0.001 and ## *p* < 0.0001 GMP vs. INDO.

Clinical manifestations of indomethacin (INDO)-induced enteropathy include decreased food intake accompanied by weight loss [36,37]. Therefore, we investigated whether GMP prevents these metabolic alterations. The average food intake at day 7, prior to initiating Na_2_CO_3_ or INDO administration, was the same among groups, with a mean intake of 13.21 ± 0.41 g. After seven days of INDO administration, a significant reduction in daily food intake was observed in animals as compared to the control (Figure 1a; *p* < 0.001). Treatment with GMP prevented the decline in food intake despite animals that were adminstered INDO (GMP group), with a mean food intake per rat of 14.36 ± 0.62 g, equivalent to that of control animals (14.88 ± 0.43 g). In relation to body weight gain, during the seven days prior to the beginning of INDO administration, it was similar among the three groups with an average gain of 14.69 ± 0.67 g. From day 8 to day 14, control animals increased their body weight by 12.11 ± 1.52%, whereas INDO-administered rats only increased by 2.85 ± 2.41% (*p* < 0.01), showing a 76.45% reduction in weight gain compared to normal controls (Figure 1b). GMP treatment significantly increased rat body weight in comparison to the INDO group (*p* < 0.0001), resulting in weight gain similar to control animals.

### 2.2. Effect of GMP on Intestinal Damage, and Weight and Length

**Figure 2:**
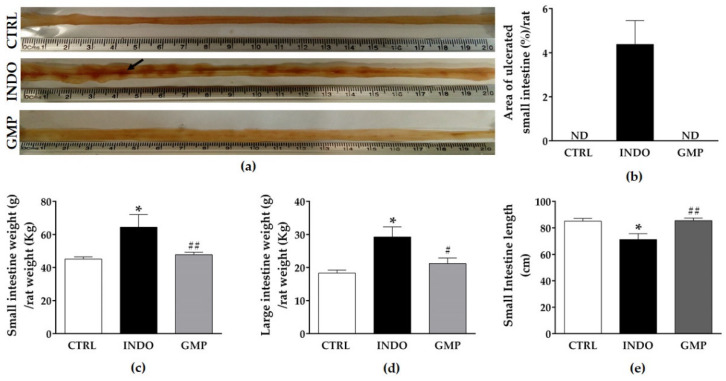
Effect of treatment with glycomacropeptide (GMP) on intestinal damage, weight and length in rats with indomethacin-induced enteropathy. (**a**) Representative pictures of the mucosal surface of the small intestine of rats from the CTRL, INDO and GMP groups. The arrow indicates ulcerative lesions. (**b**) Intestinal damage in rats presented as percentage (%) of area of ulcerated small intestine. Small (**c**) and large (**d**) intestine weights in relation to rat body weight. (**e**) Small intestine length of animals from experimental groups. Data are presented as mean ± SEM. CTRL and GMP, *n* = 14; INDO, *n* = 11. * *p* < 0.01 INDO vs. CTRL; # *p* < 0.05 and ## *p* < 0.01 GMP vs. INDO. ND: not detected.

Macroscopic observations of the small intestine showed that 6 mg/kg/day INDO administered over seven days induced severe damage (Figure 2a). The small intestine of rats from the INDO group presented with erythematous areas and multiple ulcerative lesions, while no appreciable macroscopic changes were evident in the mucosal surface of the CTRL and GMP groups. Ulcers in the INDO group were patchy in distribution, increased in number from the proximal to the distal end of the jejunum, and they were predominantly located along the mesenteric border. To make a quantitative estimation of macroscopic injury, the percentage area of ulcerated mucosa was measured (Figure 2b). INDO administration elicited ulcerative lesions on 4.38 ± 1.07% of the area of the small intestine. By contrast, GMP treatment before INDO administration resulted in the absence of macroscopically visible mucosal ulcerative erosions.

NSAID administration induces changes in the weight of gastrointestinal organs due to inflammatory process, with consequent shortening of the intestinal length [36,37]. Thus, we analyzed intestine and stomach weights expressed as a proportion of body weight, as well as net intestinal length. Significant increases in small (64.46 ± 7.60 g/kg vs. 45.10 ± 1.34 g/kg; *p* < 0.01) and large (29.25 ± 3.09 g/kg vs. 18.32 ± 0.87 g/kg; *p* < 0.01) intestine weights were evident in rats that were administered INDO compared to healthy controls (Figure 2c,d). GMP treatment restored small and large intestine weights compared to those of the control group. Besides, small intestinal length was significantly reduced in the INDO group compared to control animals; although GMP treatment prevented the shortening of the small intestine (Figure 2e). In our experimental model, there was no significant difference in stomach weight following INDO administration, recording values of 7.64 ± 0.25 g/kg in control animals versus 8.29 ± 0.39 g/kg in INDO-administered rats.

### 2.3. Effect of GMP on Hematological and Biochemical Parameters

**Figure 3:**
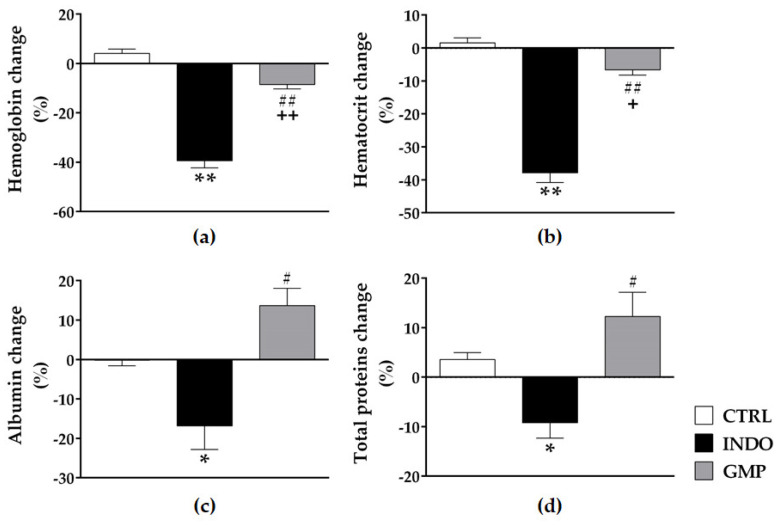
Effect of treatment with glycomacropeptide (GMP) on hematological and biochemical parameters in indomethacin-induced enteropathy. Hemoglobin (**a**) and hematocrit (**b**), as hematological variables, were measured in blood and represented as percentage (%) change at day 15 with respect to day 7. The quantity of albumin (**c**) and total proteins (**d**), as biochemical markers, was measured in serum and represented as percentage (%) change at day 15 with respect to day 7. Data are presented as mean ± SEM. CTRL and GMP, *n* = 14; INDO, *n* = 11. *p < 0.05 and ** *p* < 0.0001 INDO vs. CTRL; # *p* < 0.001 and ## *p* < 0.0001 GMP vs. INDO; + *p* < 0.01 and ++ *p* < 0.0001 GMP vs. CTRL.

Anemia is reported as a side effect of NSAID administration [38]. The INDO group resulted in a significant decrease in hemoglobin and hematocrit levels compared to the control group (Figure 3a,b; *p* < 0.0001). Animals presented with 39.35 ± 2.97% and 37.91 ± 2.86% less hemoglobin and hematocrit at day 15 than at day 7. In animals treated with GMP, the level of these hematological variables was significantly higher (*p* < 0.0001), with only 8.49 ± 1.79% and 6.62 ± 1.60% less hemoglobin and hematocrit at the end than that registered at the beginning of INDO administration, although treatment did not restore values to control levels. In addition, we evaluated two biochemical parameters related to bleeding and intestinal inflammation [38]. INDO administration considerably declined serum levels of albumin and total proteins as compared to control animals (Figure 3c,d; *p* < 0.05). As a consequence of INDO intake over seven days, animals showed 16.81 ± 5.98% and 9.19 ± 3.09% less albumin and total proteins in serum than before INDO administration. GMP treatment prevented the drop in these biochemical parameters, with 13.68 ± 4.38% and 12.27 ± 4.86% more albumin and total proteins in serum than that registered before INDO administration. Although these change percentages were slightly higher than that of the control group, they were within the normal range and there were non-significant differences between GMP treated and control rats, showing that GMP is protective against the decay of albumin and total proteins prompted by INDO administration. We also evaluated alanine aminotransferase (ALT) serological levels to discard liver damage or failure in our animal model of INDO-induced enteropathy and they were similar in the three rat groups, with a mean value of 43.53 ± 0.96 U/L.

### 2.4. Effect of GMP on Intestinal Inflammation

**Figure 4:**
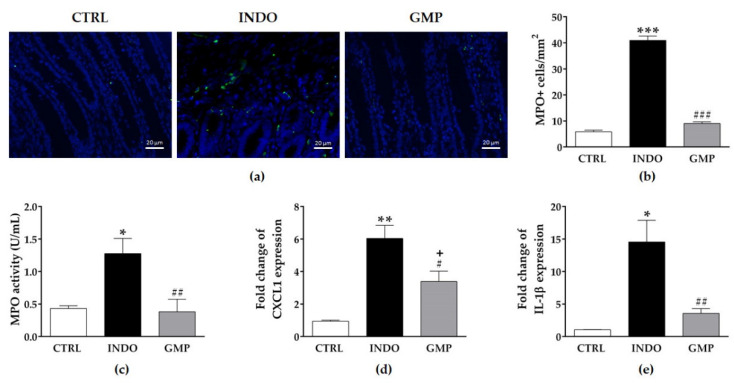
Effect of treatment with glycomacropeptide (GMP) on intestinal inflammation in rats with indomethacin-induced enteropathy. (**a**) Immunofluorescence staining of myeloperoxidase (MPO) positive cells (green) in the intestinal mucosa of rats from the CTRL, INDO and GMP groups (scale bar = 20 µm). The quantity of MPO+ cells per intestinal area unit (**b**) and MPO activity (**c**) in intestinal tissue. Intestinal expression level of CXCL1 (**d**) and interleukin (IL)-1β (**e**) relative to β-actin was analyzed by qPCR and presented as fold change. Data are presented as mean ± SEM. CTRL and GMP, *n* = 14; INDO, *n* = 11. * *p* < 0.01, ** *p* < 0.001 and *** *p* < 0.0001 INDO vs. CTRL; # *p* < 0.05, ## *p* < 0.01 and ### *p* < 0.0001 GMP vs. INDO; + *p* < 0.05 GMP vs. CTRL.

Neutrophil infiltration plays a crucial role in NSAID-induced enteropathy [20,39]. The amount of myeloperoxidase (MPO) positive cells and MPO activity in intestinal mucosa were used as indicators of neutrophil infiltration, and hence of acute inflammation [40]. Histological analysis revealed that INDO administration elicited the infiltration of MPO positive cells into the lamina propria, but GMP treatment attenuated the number of infiltrated neutrophils (Figure 4a). Morphological assessment showed that the number of MPO positive cells in the intestinal mucosa of INDO administered rats was 7.02 times higher than in control animals (40.97 ± 1.66 cells/mm^2^ vs. 5.83 ± 0.66 cells/mm^2^, *p* < 0001; Figure 4b). Whereas in animals treated with GMP, the number of these inflammatory cells per mm^2^ was reduced to 9.02 ± 0.63 cells/mm^2^ (*p* < 0.0001). Equally, MPO activity in the intestinal mucosa from INDO-administered rats increased 2.96-fold compared to control animals (1.27 ± 0.23 U/mL vs. 0.43 ± 0.04 U/mL, *p* < 0.01; Figure 4c). However, MPO activity was remarkably lowered to 0.38 ± 0.19 U/mL by GMP treatment (*p* < 0.01). As CXCL1 is a chemotactic factor involved in neutrophil recruitment [41], we investigated the mRNA expression level of this chemokine in mucosal tissue. In accordance with neutrophil infiltration, we found that CXCL1 gene expression was 6.37-fold higher in INDO-administered rats than in the control group (*p* < 0.001), and that GMP treatment significantly reduced its expression (Figure 4d; *p* < 0.05). We also analyzed the mRNA expression change of IL-1β, one of the inflammatory cytokines involved in NSAID-induced enteropathy [42]. As shown in Figure 4e, the expression of IL-1β was 13.88-fold higher in INDO-administered than in control animals (*p* < 0.01), but it was clearly decreased by GMP treatment (*p* < 0.01).

### 2.5. Effect of GMP on Intestinal Oxidative Damage

**Figure 5:**
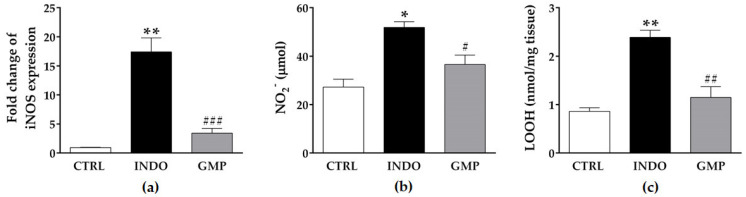
Effect of treatment with glycomacropeptide (GMP) on intestinal oxidative damage in rats with indomethacin-induced enteropathy. (**a**) Intestinal expression level of inducible nitric oxide synthase (iNOS) relative to β-actin is presented as fold change. The quantity of nitrites (NO_2_^−^) (**b**) and lipid hydroperoxides (LOOH) (**c**) in intestinal tissue was evaluated by Griess reaction and ferric-xylenol orange method, respectively. Data are presented as mean ± SEM. CTRL and GMP, *n* = 14; INDO, *n* = 11. * *p* < 0.001 and ** *p* < 0.0001 INDO vs. CTRL; # *p* < 0.05, ## *p* < 0.001 and ### *p* < 0.0001 GMP vs. INDO.

Inducible nitric oxide synthase (iNOS) activation and nitric oxide (NO) production are involved in the pathogenesis of NSAID-induced intestinal lesions [20]. Therefore, we investigated whether GMP suppresses these oxidative factors in the enteropathy induced by NSAIDs. In rats receiving oral INDO, iNOS gene expression and nitrite level in intestinal tissue were 18.19- and 1.9-fold higher than in the control group (Figure 5a,b; *p* < 0.0001 and *p* < 0.001, respectively). Prophylaxis with GMP significantly reduced both oxidative markers. As an index of oxidative damage, we determined membrane lipid peroxidation (Figure 5c). The value of lipid hydroperoxides (LOOH) in the intestinal mucosa of control rats was 0.86 ± 0.07 nmol/mg tissue, but INDO administration significantly increased it to 2.38 ± 0.14 nmol/mg tissue (*p* < 0.0001). GMP treatment reduced the tissue accumulation of INDO-induced LOOH to 1.14 ± 0.22 nmol (*p* < 0.001), restoring values to those of control animals.

### 2.6. Effect of GMP on Mucosal Barrier Integrity

**Figure 6:**
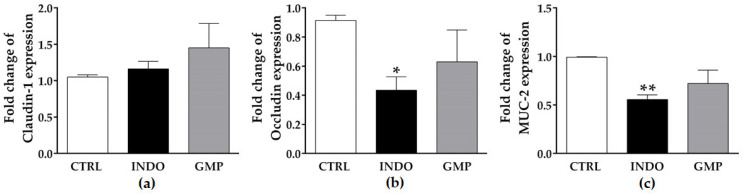
Effect of treatment with glycomacropeptide (GMP) on mucosal barrier integrity in rats with indomethacin-induced enteropathy. Intestinal expression level of claudin-1 (**a**), occludin (**b**) and mucin (MUC)-2 (**c**) relative to β-actin was analyzed by qPCR and presented as fold change. Data are presented as mean ± SEM. CTRL and GMP, *n* = 14; INDO, *n* = 11. * *p* < 0.05 and ** *p* < 0.01 INDO vs. CTRL.

Mucosal barrier function is disrupted due to NSAID-induced PG deficiency and mitochondrial malfunction [39]. We measured the mRNA expression of two tight junction components and mucin in the small intestine of animals from the three experimental groups. No change was shown in claudin-1 expression in the intestinal tissue of INDO-administered animals (Figure 6a), while occludin and mucin (MUC)-2 expression were reduced by approximately half (Figure 6b,c; *p* < 0.05 and *p* < 0.01, respectively). GMP treatment induced a slight increase in occludin and MUC-2 expression, although it was not significant.

### 2.7. Effect of GMP on Survival Rate

**Figure 7:**
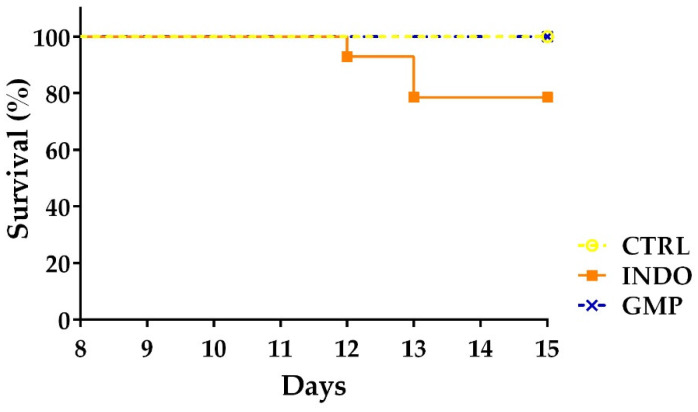
Effect of treatment with glycomacropeptide (GMP) on the survival of rats with indomethacin-induced enteropathy. Data as presented as percentage (%) of animal survival each day of the damage-induction protocol in the CTRL, INDO and GMP groups (*n* = 14 at day 8).

Finally, we analyzed animal survival during the time of INDO administration using a Kaplan–Meier curve (Figure 7). The survival of the INDO group was 100% during the first four days (day 8 to 11), 92.85% at day 12 and 78.57% from day 13 to the final day of the study. Strikingly, GMP treatment avoided animal death, registering survival data of 100%, similar to the control group.

## 3. Discussion

The prevalence of enteropathy due to NSAID consumption is growing, while the rate of upper gastroduodenal damage is decreasing [11]. The latter is due to the concomitant use of proton pump inhibitors in patients with a high risk of gastroduodenal events. Nevertheless, recent scientific evidence shows that these inhibitors exacerbate intestinal damage [43,44,45]. Patients with lower gastrointestinal complications have longer stays in hospital, require more resources, and present with increased mortality than those affected by gastroduodenal damage [46]. An additional consideration is that most of the patients with NSAID enteropathy are asymptomatic, which has been recently demonstrated using wireless capsule enteroscopy [47]. As there is not an approved therapeutic regimen to treat or prevent NSAID-induced enteropathy, it is crucial that experimental studies look forward to potential treatments that could prevent this pathological condition.

Recently, food-derived peptides have been considered as effective alternatives to drugs. In this study we investigated whether GMP, a bioactive peptide obtained from milk κ-casein, may protect against enteropathy induced by indomethacin. The results of our study demonstrated that oral administration of GMP strongly impacts on metabolic, hematological and biochemical changes associated with the clinical symptomatology, by avoiding the drop in food intake and body weight, and by increasing hemoglobin, hematocrit, albumin and total protein levels in animal blood. Besides, GMP administration prevents the development of ulcerative lesions in the small intestine and increases animal survival. Our study also provides additional evidence for the major preventive properties of GMP on NSAID enteropathy. GMP targets the underlying intestinal inflammatory and oxidative responses, as it decreases neutrophil infiltration, IL-1β, CXCL1, and iNOs mRNA expression, as well as LOOH and NO levels.

We used a rat model to study a potential treatment to indomethacin-induced enteropathy, as it is reproducible and similar to the human condition in relation to clinical manifestations and factors involved in pathology development [18]. In our work, a daily dose of 6 mg/kg of indomethacin over 7 days was employed, resulting in a reduction of food intake accompanied by weight loss. Similar metabolic changes are reported in humans and in rat models of intestinal damage by NSAIDs [36,43,48,49,50]. The GMP treatment avoided the decrease in food intake and the loss of body weight. Accordingly, in animal models of trinitrobenzenesulfonic acid (TNBS)- and famozadone (OXZ)-induced colitis, GMP administration decreased anorexia and increased weight gain in animals [31,51]. The reduction in food intake due to indomethacin is related to increased levels of systemic proinflammatory cytokines [52]. Along this line, feeding phenylketonuric mice with a GMP diet prevents systemic inflammation and decreases plasma levels of inflammatory cytokines [53].

In agreement with previous data [36], we observed that indomethacin induces an increase in intestine weight, likely as a result of the inflammatory process. Inflammation is characterized by hyperemia, edema, and inflammatory cell infiltration (mainly neutrophils) [16,20,54]. GMP treatment significantly prevented the augment in intestine weight, which is in accordance with the decrease in the number of MPO positive cells infiltrated into intestinal tissue. No change was observed in stomach weight, indicating that the inflammatory process was mainly at the intestinal level. Small intestine length was reduced as a result of indomethacin administration. Intestine shortening is reported as a compensatory mechanism to increase mucosal surface area in response to intestinal damage [55]. GMP treatment prevented the shortening of the small intestine, which coincides with the absence of macroscopically ulcerative lesions.

Anemia is included among the clinical manifestations of NSAID enteropathy and is related to blood loss or the impaired absorption of iron [56,57,58]. Small intestine inflammation caused by NSAIDs is also associated with hemorrhage [59]. Our rats showed a decrease in hemoglobin and hematocrit level in blood after indomethacin administration. GMP prophylaxis decreased the drop in these hematological parameters, indicating that the treatment is ameliorating the hemorrhage and anemia in the animals. Patients with NSAID-induced small intestine inflammation are also found to have a protein-losing enteropathy [59]. The decline in serum albumin and total protein induced by NSAIDs are regarded as indices of intestinal vascular leakage. Previous studies report that serum albumin is a readily measured endogenous parameter of acute intestinal leakage in NSAID-induced enteropathy [60]. In our study, indomethacin caused a marked decrease in albumin and total protein levels in serum without affecting serum biochemical markers of liver function, but GMP treatment prevented hypoalbuminemia and hypoproteinemia. These results are in agreement with the findings of Kim and coworkers [61], who showed that bovine colostrum administration, an abundant source of GMP, reduces protein-losing enteropathy induced by diclofenac in mice.

The integrity of the intestinal barrier is crucial for the maintenance of homeostasis. When the production of tight junction proteins is compromised, the loss of connection among intestinal epithelial cells increases the permeability of the intestinal epithelium [62,63]. In NSAID enteropathy, an initial increase in intestinal permeability is a prerequisite for the subsequent development of small intestine inflammation [64]. As documented by Shi and collaborators [65], indomethacin administration in rats induces a downregulation of tight junction proteins, occludin and zonula occludens (ZO)-1 in intestinal tissue. Besides, human gastric epithelial cells (MKN-28) exposed to indomethacin reduce the expression of occludin without affecting that of ZO-1 and claudin-1 [66]. Along this line, we confirmed that the mRNA expression of occludin is significantly lower in our indomethacin-induced enteric damage model, with no change in claudin-1 expression. In addition, MUC-2 production is also diminished in our rat model, indicating that the intestinal mucosal barrier is compromised by indomethacin administration, as previously reported [50]. GMP treatment did not recover the reduction in mRNA expression of these genes involved in barrier function, although it positively impacted the subsequent inflammatory response.

NSAID enteropathy is characterized by a massive infiltration of neutrophils in ulcerated areas [67]. Thus, the level of fecal calprotectin, a non-degraded neutrophil cytosolic protein, is used as a biomarker of intestinal damage in humans [68]. In addition to neutrophil migration, MPO activity is another important marker of possible damage in inflamed tissue [67,69]. Our results showed an increased number of infiltrating neutrophils, as well as augmented MPO activity, in the intestinal tissue of rats administered indomethacin. Ulcerative lesions were also present in the small intestine mucosa. GMP treatment strongly decreased the number of these inflammatory cells and the MPO activity, and prevented the development of ulcers by indomethacin in the small intestine. In accordance, the therapeutic effect of GMP on MPO activity and intestinal damage extension in TNBS-induced ileitis has been reported [32]. Our findings indicate that the beneficial effect of GMP is mediated by the attenuation of the intestinal inflammatory process, as reflected by the significant reduction in mRNA expression of CXCL1 and IL-1β. CXCL1 is regarded as the rodent functional homologue of the human neutrophil chemokine IL-8, and has been found to contribute to the pathology of a number of neutrophil dependent animal models of disease [41]. On the other hand, Higashimori and coworkers [42] reported that the administration of indomethacin induces the expression of IL-1β in mice, which is accompanied by an increased lesion index in the intestine. Along this line, a pharmacological inhibitor of IL-1β production is able to reduce the severity of intestinal lesions induced by indomethacin in rats [20]. All together, these results indicate that GMP may protect against the development of NSAID enteropathy by the downregulation of intestinal inflammation. Negative regulatory effects of GMP on IL-1β gene expression have been reported in other intestinal disease experimental models, such as ileitis and colitis [32,33].

Another important fact to consider is that intestinal microbiota and mast cells are crucial elements in the initiation of the inflammatory response and mucosal damage [39,70]. Gnotobiotic rats mono-associated with *Bifidobacterium* or *Lactobacillus* have no ulcers after NSAID administration, while mono-association with *Escherichia coli* or *Eubacterium limosum* induces ileal ulcer formation [71]. On the other hand, the development of intestinal lesions in response to indomethacin is prevented in mast cell-deficient rats [72]. It has been shown that orally administered GMP induces an increment in the amount of *Lactobacillus* and *Bifidobacteria* in the fecal microbiota of mice [73], and that this bioactive peptide avoids the adhesion of enterohemorrhagic *E. coli* O157:H7 on Caco-2 cells [74]. Moreover, we previously demonstrated that GMP is able to modulate mast cell activation [75]. In this context, a protective role of GMP on intestinal damage through its prebiotic activity, or by avoiding the adhesion of detrimental bacteria, or the activation of mast cells should not be disregarded. Direct noxious topical effects of NSAIDs on enterocytes and massively infiltrated neutrophils are the main sources of free radicals during enteropathy, causing oxidative damage in intestinal mucosa [13,14,19,20]. In the present study, we demonstrated that increased iNOS mRNA expression and NO production are associated with respiratory burst in the intestine of enteric-damaged rats, which increases the lipid peroxidation as a consequence. At the same time, neutrophil infiltration and ulcer formation is highly promoted in our animals. Konaka and collaborators show that iNOS activity and NO production is temporally correlated with the occurrence of intestinal damage in rats after indomethacin administration [20]. When they delete neutrophils or use an IL-1β inhibitor in animals, the severity of intestinal lesions and the increase in iNOS activity are reduced. These findings are consistent with the results of our study, as GMP treatment reduced oxidative damage in intestinal tissue parallel to the decrease in inflammatory markers and intestinal lesions. We suggest that the antioxidant activity of GMP is participating in the protective effect on indomethacin enteropathy. A downregulatory effect of GMP on iNOS gene expression has been previously reported in TNBS-induced experimental models of intestinal damage [31,32]. Overall, our results demonstrate that the GMP protective effect relies on its antioxidant and anti-inflammatory properties.

It is important to highlight that different GMP products have been used to demonstrate the biological activities of GMP in animal models of diverse pathologies [29,30,31,32,33]. As several of the bioactive properties ascribed to GMP in vitro, such as antibacterial, prebiotic and immune cell-proliferating activities, are caused by carbohydrate molecules (mainly sialic acid [76]), the evaluation of sugar content on GMP products may be crucial to reproduce a biological response. Besides, upon GMP ingestion, some peptides with biological activities can be released. In this context, a GMP hydrolysate has been shown to decrease NO production, iNOS and IL-1β expression, as well as the activation of the transcription factor NF-κB in LPS-activated macrophages, the latter regulating the expression of many inflammatory genes [35]. The same hydrolysate has been reported to increase the activation of the nuclear factor Nrf2, a key regulator of the antioxidant response, and to decrease reactive oxygen species production in damaged HepG-2 cells and macrophages [77,78]. As indomethacin causes a decrease in Nrf2 activation in rat gastric mucosa and activates the NF-κB pathway and IL-8 production in Caco-2 cells [79], derived peptides from GMP hydrolysis may be also directly modulating the antioxidant or anti-inflammatory activity on NSAID-enteropathy. The limitation of this study is a lack of in-depth exploration of possible molecular targets and cell signaling pathways regulated by GMP.

## 4. Materials and Methods 

### 4.1. Experimental Animals

Forty-two male Wistar rats (8 weeks old, 120–150 g) were obtained from the Laboratory Animal Service from the Autonomous University of Aguascalientes. Animals were maintained under controlled conditions of temperature (20–22 °C) and illumination (12 h periods of light/darkness). Food (Rodent Laboratory Chow 5001; Purina, Mexico) and water were freely accessible. Rats were housed in metallic cages (3–4 animals/cage) with adequate sawdust substrate. Experimental protocols in this study were approved by the Institutional Ethical Committee for the Use of Animal in Teaching and Research (approval date: 04/10/2017). All participants and animal handlers were committed to carefully manipulating the rats in order to reduce stress and suffering.

### 4.2. Indomethacin-Induced Enteropathy Model and Sample Collection

**Figure 8:**
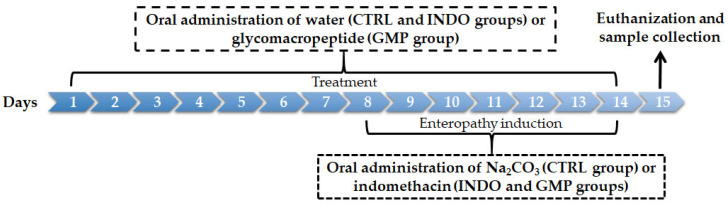
Schematic diagram of experimental protocol for indomethacin-induced enteropathy and glycomacropeptide (GMP) administration.

Animals received enrofloxacin (0.06%) in drinking water for three days, and then acclimatized for seven days. Later, rats were randomly assigned to the study groups (*n* = 14 rats/group): i) control group (CTRL; animals receiving Na_2_CO_3_ 5% orally and water); ii) INDO group (animals were administered indomethacin and treated with water); and iii) GMP group (animals were administered indomethacin and treated with GMP). For enteropathy induction, animals in the INDO and GMP groups received indomethacin at 6 mg/kg/day in a solution of Na_2_CO_3_ 5% as vehicle over 7 days, while the CTRL group was administered 1 mL of Na_2_CO_3_ 5% (Figure 8). Treatment was administered 7 days prior for enteropathy induction, and dispensed 4 h previous to indomethacin or Na_2_CO_3_ administration. For the GMP group, animals were treated orally with 500 mg/kg/day GMP (Lacprodan cGMP-10, gifted by Arla Food Ingredients Group P/S, Viby, Denmark) dissolved in tap water; while for the CTRL and INDO groups, animals received 1 mL of tap water orally/day. All compounds or vehicles were administered using an esophageal catheter. According to the manufacturer, Lacprodan cGMP-10 contains 74% GMP, 2% fat, 0.5% lactose, and 4.2% sialic acid. The dose of GMP used in this work was established based on previous therapeutic studies in rats [29,30,31,32,33]. The dose of indomethacin (6 mg/kg/day) was determined from a previous study we developed for this purpose in Wistar rats. Indomethacin dosage (4 mg/kg/day, 6 mg/kg/day, 8 mg/kg/day, and 10 mg/kg/day) were tested and the 6 mg/kg/day dose seemed to be the most appropriate for the current investigation based on survival rate, ulcerated small intestine area, and tissue consistency (Figure S1).

At day 15, and after 8 h of fasting, rats were euthanized by anesthesia overdose. Blood samples were taken from caudal or abdominal aorta veins at days 7 and 15, respectively. The stomach and small and large intestines were excised, washed with phosphate-buffered saline (PBS, pH 7.4), and weighed. In relation to the small intestine, it was measured from the ileocecal to the pyloric valve. Four tissue segments of 1 cm were recovered from the distal jejunum for posterior analysis. The first one was collected and stored at −80 °C in RNAlater (Ambion, Austin, TX, USA) for the evaluation of mRNA expression, the second one was immersed in lysis buffer for the quantification of nitrites and LOOH, the third segment was immersed in buffer to analyze MPO activity, and the last one was fixed in neutral formalin for histological analysis. Remaining small intestine tissue segments were used to evaluate macroscopic damage.

### 4.3. Changes in Food Intake and Body Weight

Food intake of the rats and their body weights were measured every morning. The weight of the food placed each day in rat cages was measured and the food intake was calculated by subtracting the weight of uneaten food the next morning and dividing by the number of rats in the cage. The percentage change in body weight was calculated by comparing rat weight at day 14 with that at day 7.

### 4.4. Macroscopic Assessment of Small Intestine Damage

The small intestine was opened longitudinally along the anti-mesenteric border, washed with cold PBS, fixed in 10% neutral buffered formalin for 24 h, rinsed, and transferred to 70% ethanol for 30 min. The intestinal internal surface was visualized and all ulcerative lesions were quantified and measured with a digital Vernier caliper (Milomex, Ltd, Bedfordshire, UK) to determine their area. Intestinal damage was expressed as a percentage of ulcerated area of the total intestinal segment surface.

### 4.5. Evaluation of Hematological and Biochemical Parameters

For hemoglobin and hematocrit quantification, 200 µL of blood collected in heparinized tubes was evaluated in an automated hematologic analyzer Orphée Mythic 18 (Diamond Diagnostics, Holliston, MA, USA). Biochemical parameters, such as albumin, total protein, and ALT, were determined in serum from blood collected in non-heparinized tubes. The study was performed in an automated clinical chemistry analyzer Vitros 5600 Integrated System (Ortho Clinical Diagnostics, Raritan, NJ, USA).

### 4.6. Myeloperoxidase Activity

To evaluate the neutrophil infiltration due to inflammatory processes, the MPO activity in intestinal tissue was quantified by the method described by Bradley [40]. Briefly, a 1 cm segment of intestine was homogenized in hexadecyltrimethylammonium bromide 0.5% (HTAB) in potassium phosphate buffer (50 mM, pH 6.0) with an Ultra-Turrax T10 homogenizer (Ika, Wilmington, NC, USA) and centrifuged at 510 g for 10 min at 4 °C. Supernatant (40 µL) was mixed with 200 µL of *O*-dianisidine dihydrochloride 0.167 mg/mL and hydrogen peroxide 0.0003% in phosphate buffer (50 mM, pH 6.0). The change in absorbance was measured at 450 nm in an iMark microplate reader (Biorad, Hercules, CA, USA). For MPO activity determination, one unit of MPO was defined as the degradation of 1 µmol of hydrogen peroxide per minute at 25 °C.

### 4.7. Immunofluorescence Assay

Fixed intestine tissue was paraffin embedded and 4–5 µm sections were mounted on silanized slides. After deparaffination and rehydratation, antigen retrieval was carried out by heating the slides in a pressure cooker in citrate buffer (citric acid 10 mM, sodium citrate 10 mM, pH 6.0) for 10 min. Sections were blocked for 30 min with fetal bovine serum in PBS with 0.2% triton. After washing, slides were incubated at room temperature with a polyclonal rabbit anti-MPO antibody (diluted 1:50; Abcam, Cambridge, MA, USA) overnight. Slides were washed and incubated with an Alexa 488 conjugated goat anti-rabbit IgG (diluted 1:1000; Invitrogen, Carlsbad, CA, USA) for 2 h. Nuclei were counterstained with Hoechst solution and slices were mounted. Sections were visualized in an AxioPlan Carl Zeiss Microscope (Zeiss, Oberkochen, Germany) at a magnification of 40× and digitalized with the AxioVision Rel 4.8 software (Zeiss). MPO-positive neutrophils were counted in three slices/animal and five animals/group, and reported as the number of cells per unit of area (mm^2^).

### 4.8. Determination of Nitric oxide and Lipids Hydroperoxides

The segment of intestine was washed with PBS, homogenized in 350 µL of tris-HCl (20 mM, pH 7.4) with an Ultra-Turrax T10 homogenizer (Ika), and clarified by centrifugation for 10 min at 10,000× *g* and 4 °C. Proteins were precipitated from 200 µL of supernatant by mixing with 400 µL of ammonium sulfate 5% for 15 min on ice. Sample was centrifuged for 20 min at 3000× *g* and 4 °C. Supernatant was recovered and centrifuged for 20 min at 3000× *g* and 4 °C. Obtained supernatant was employed for assessment of NO and LOOH.

Due to instability of NO molecule, Griess assay was employed to detect the amount of nitrite, a stable NO metabolite. For Griess assay (adapted from [80]), 100 µL of sample was mixed with 50 µL of sulfanilic acid 1% in H_3_PO_4_ 5% and 50 µL of alpha-Naphthylamine 0.5% in acetic acid 5 N. The absorbance of chromophore formed due diazotization between nitrite and sulfanilic acid and coupling of alpha-Naphthylamine was measured at 490 nm in SmartSpec Plus spectrophotometer (BioRad). Nitrite concentration was quantified with a standard curve of serial 1:2 dilutions of sodium nitrite 100 µM prepared in Tris-HCl (20 mM, pH 7.4). Standards were processed as mentioned for samples.

Lipid peroxidation is the result of oxidative stress on cell membranes, and its quantification was addressed by the ferric-xylenol orange method [81,82,83]. For this reaction, 45 µL of sample was mixed with 55 µL of methanol 90% and incubated at room temperature for 30 min. Then, 900 µL of FOX2 reagent (ferrous oxidation in xylenol orange, version 2) were added and incubated for 30 min. FOX2 reagent was prepared from two solutions: solution A comprised ammonium ferrous sulfate 250 µM in sulfuric acid 25 mM, and solution B contained xylenol orange 100 µM and butylated hydroxytoluene 4 mM, both solutions prepared in 90% *v/v* methanol. Absorbance was measured at 560 nm in a SmartSpec Plus spectrophotometer (BioRad). Quantification of LOOH was established with a standard curve of tert-Butyl hydroperoxide from 1 to 16 nM in 90% *v/v* methanol.

### 4.9. RNA Extraction, Retrotranscription and qPCR

Total RNA was extracted from 100 mg of the corresponding intestinal tissue with the GeneJET RNA Purification Kit (Thermo Scientific, Waltham, MA, USA) and treated with RQ1 RNase-Free DNase (Promega, Madison, MI, USA) and then quantified with a Nanodrop 2000 (Thermo Scientific). For cDNA synthesis, reverse transcription was performed using the First Strand cDNA Synthesis Kit (Thermo Scientific) with the oligo(dT)18 in a 2720 Thermal Cycler (Applied Biosystems, Foster City, CA, USA) according to the manufacturer´s protocol. Quantitative real-time PCR was established with the Maxima SYBR Green/ROX qPCR (Thermo Scientific) in a StepOne Real-Time PCR System (Applied Biosystems). Primers used for the quantification of mRNA expression are listed in Table 1. Expression levels were determined in five animals per group with the 2^−∆∆Ct^ method using β-actin as a housekeeping gene [84] and represented as fold change.

### 4.10. Statistical Analysis

All data are represented as the mean ± standard error of the mean (SEM). Statistical analyses were performed using the GraphPad Prism v.5.01 software (GraphPad Software Inc., La Jolla, CA, USA). Comparisons among groups were made with the two-way analysis of variance (ANOVA) test with the Bonferroni *post hoc*. Significance was set at *p* < 0.05.

## 5. Conclusions

This study provides strong evidence of the beneficial effect of GMP on NSAID-enteropathy, which is related to anti-inflammatory and antioxidant activities at the intestinal level. These findings suggest that GMP could be a novel natural agent to prevent intestinal damage in patients using NSAIDs. Ongoing research continues to explore the effect of GMP in the modulation of microbiota composition during NSAID administration, and to understand the exact cellular and molecular mechanism by which intact or hydrolyzed GMP protects against enteric damage, with special focus in NF-κB and Nrf2 pathways.

## Figures and Tables

**Table 1:** Primers used in this study.

Target	Oligonucleotide	Accession Number (NCBI)
Claudin-1	Fw: AACCTCTTACCCAACACCACG Rv: GCCAAGACCCTCATAGCCAT	NM_031699.2
Occludin	Fw: AGGACAGACCCAGACCACTA Rv: ACTCTTCGCTCTCCTCTCTG	NM_031329.2
MUC-2	Fw: GTATGTGCTCGCCTGTATGC Rv: TGACCTCCAGATGTGAGCAG	XM_017604244.1
CXCL1	Fw: GCACCCAAACCGAAGTCATAG Rv: TGTTGTCAGAAGCCAGCGTT	NM_030845.1
IL-1β	Fw: AAATCTCACAGCAGCATCTC Rv: ACTAGCAGGTCGTCATCATC	NM_031512.2
iNOS	Fw: GATGTGCTGCCTCTGGTCCT Rv: ACTCCAATCTCGGTGCCCAT	NM_012611.3
β-Actin	Fw: GTCGTACCACTGGCATTGTG Rv: GCTGTGGTGGTGAAGCTGTA	NM_031144.3

NCBI, National Center for Biotechnology Information.

## Supplementary Materials

The following are available online, Figure S1: Determination of indomethacin dose to induce enteropathy model in Wistar rat.
