# Supplementary material for: Glycomacropeptide Ameliorates Indomethacin-Induced Enteropathy in Rats by Modifying Intestinal Inflammation and Oxidative Stress
Source: Molecules. 2020 May 18;25(10):2351. doi: 10.3390/molecules25102351 (PMC7287897; doi:10.3390/molecules25102351)
Supplement: Supplementary file 1 [file molecules-25-02351-s001.pdf]

Supplementary Materials: The following are available online at www.mdpi.com/xxx/s1, Figure S1: Determination of indomethacin dose to induce enteropathy model in Wistar rat.

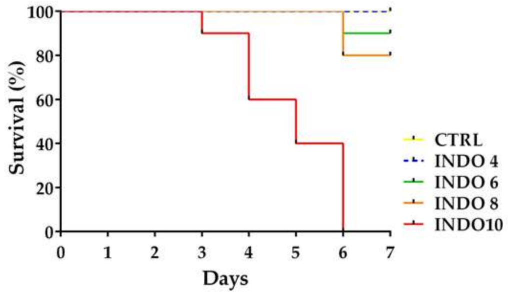

(a)

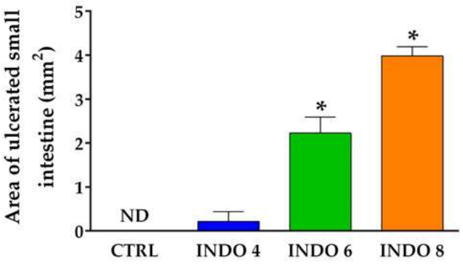

(b)

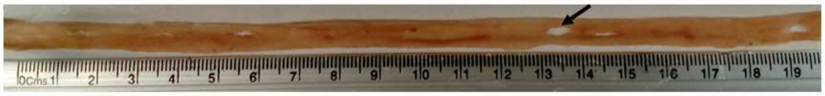

(c)

Figure S1. Determination of indomethacin dose to induce enteropathy model in Wistar rat. Indomethacin dosage (4, 6, 8, and 10 mg/kg/day) were tested in Wistar rats divided into 5 groups: CTRL, INDO 4, INDO 6, INDO 8, INDO 10. (**a**), survival rate of animals using a Kaplan-Meier curve. The 10 mg/kg/day dose of indomethacin drastically reduced the survival of rats to 0% at day 6. For doses of 4, 6 and 8 mg/kg/day of indomethacin survival rate reached the 100, 90 and 80% at day 6, respectively. Data as presented as percentage (%) of animal survival each day of damage-induction protocol. Postmortem observations showed that the small intestine of animals administered with 10 mg/kg/day of indomethacin was completely damaged, even making impossible to determine the quantity of ulcers. (**b**), ulcerated area of small intestine of animals administered with 4, 6 and 8 mg/kg/day of indomethacin occupied small (0.21 mm^2), medium (2.22 mm^2) and big (3.98 mm^2) surfaces of tissue, respectively. (**c**), small intestine from animals receiving the 8 mg/kg/day dose had many perforations that made difficult tissue handling due to its brittle consistency. Arrow indicates a perforation. Data are presented as mean ± SEM. $n = 12$. *$p < 0.001$ INDO 6 vs. INDO 4 and INDO 8 vs. INDO 6.
